# Survival benefit of active surveillance for papillary thyroid carcinoma: a propensity score matching analysis based on SEER database

**DOI:** 10.3389/fonc.2023.1185650

**Published:** 2023-06-09

**Authors:** Jinzhe Bi, Peng-fei Lyu, Yu Wang, Hao Zhang

**Affiliations:** ^1^ Department of General Surgery, The First Affiliated Hospital of Hainan Medical University, Haikou, China; ^2^ Department of Breast Surgery, The First Affiliated Hospital of Hainan Medical University, Haikou, China

**Keywords:** papillary thyroid microcarcinoma (PTMC), active surveillance (AS), papillary thyroid carcinoma (PTC), propensity score matching (PSM), SEER (Surveillance Epidemiology and End Results) database

## Abstract

**Background:**

Over-treatment of papillary thyroid microcarcinoma (PTMC) has become a common issue. Although active surveillance (AS) has been proposed as an alternative treatment to immediate surgery for PTMC, its inclusion criteria and mortality risk have not been clearly defined. The purpose of this study was to investigate whether surgery can achieve significant survival benefits in patients with larger tumor diameter of papillary thyroid carcinoma (PTC), in order to evaluate the feasibility of expanding the threshold for active surveillance.

**Methods:**

This study retrospectively collected data of patients with papillary thyroid carcinoma from the Surveillance, Epidemiology, and End Results (SEER) database between 2000 and 2019. The propensity score matching (PSM) method was used to minimize confounding factors and selection bias between the surgery and non-surgery groups, and to compare the clinical and pathological characteristics between the two groups based on the SEER cohort. Meanwhile, the impact of surgery on prognosis was compared using Kaplan-Meier estimates and Cox proportional hazard models.

**Results:**

A total of 175,195 patients were extracted from the database, including 686 patients who received non-surgical treatment, and were matched 1:1 with patients who received surgical treatment using propensity score matching. The Cox proportional hazard forest plot showed that age was the most important factor affecting overall survival (OS) of patients, while tumor size was the most important factor affecting disease-specific survival (DSS) of patients. In terms of tumor size, there was no significant difference in DSS between PTC patients with tumor size of 0-1.0cm who underwent surgical treatment and those who underwent non-surgical treatment, and the relative survival risk began to increase after the tumor size exceeded 2.0cm. Additionally, the Cox proportional hazard forest plot showed that chemotherapy, radioactive iodine, and multifocality were negative factors affecting DSS. Moreover, the risk of death increased over time, and no plateau phase was observed.

**Conclusion:**

For patients with papillary thyroid carcinoma (PTC) staged as T1N0M0, AS is a feasible management strategy. As the tumor diameter increases, the risk of death without surgical treatment gradually increases, but there may be a threshold. Within this range, a non-surgical approach may be a potentially viable management strategy. However, beyond this range, surgery may be more beneficial for patient survival. Therefore, it is necessary to conduct more large-scale prospective randomized controlled trials to further confirm these findings.

## Introduction

The incidence of thyroid cancer is increasing worldwide (1), and the main reason for the increase in thyroid cancer incidence is the rising number of diagnoses of papillary thyroid carcinoma (PTC) (2, 3). In addition, Papillary thyroid carcinoma is the most prevalent form of malignant thyroid tumors, making up around 90% of all cases (4). The World Health Organization (WHO) classifies papillary thyroid carcinoma with a diameter of ≤1.0 cm as micro papillary thyroid carcinoma (5). Currently, some studies focus on the clinical and pathological differences between PTC and PTMC, as well as whether surgery is the preferred treatment method for all PTC patients. Due to the treatability and relatively good survival rate of papillary thyroid carcinoma, it is called an “indolent cancer”. Especially for PTMC, the vast majority of cases do not pose a threat to the patient’s life, therefore, an alternative treatment approach called active surveillance (AS) has emerged in the past decade (6–8). AS is an active management approach that has curative potential. Surgery is temporarily deferred until evidence of clear disease progression is found. AS differs from watchful waiting, which is a palliative approach that lacks active treatment and symptom monitoring, and is relatively passive in its management. Instead, AS is based on the assumption that delaying initial diagnosis and treatment will not adversely affect the disease prognosis. The main clinical benefit of AS is that it allows thousands of patients with papillary thyroid microcarcinoma (PTMC) to avoid unnecessary surgery and radioactive iodine therapy each year. In 2010, Japan included AS as a clinical guideline for monitoring (9). In 2015, the American Thyroid Association (ATA) recommended considering AS for low-risk PTMC, these low-risk factors mainly refer to PTMC that shows no invasion of surrounding tissues, no metastasis, and no evidence of high aggressiveness based on cellular or molecular markers (10). Although AS is feasible, it has not been widely implemented so far because the majority of PTMC patients have undergone thyroid surgery (11). The difficulties in implementing AS include physician reluctance, patient anxiety, and the need for well-validated selection criteria. The selection criteria for AS are based on tumor size and progression parameters (i.e., a 10mm size limit and 3mm growth limit), which are necessarily conservative for establishing safety, as such thresholds may not translate into actual mortality risk. The 10mm diameter threshold for PTMC is somewhat arbitrary, as biologically, larger diameter PTCs also exhibit clinical indolence. It is worth noting that in the United States, as the incidence of thyroid cancer increases, 87% of cases are caused by PTC with a diameter ≤20mm^3^. Therefore, there are still many unknowns about which patients represent ideal candidates for active surveillance. Survival analysis of thyroid papillary carcinoma patients who undergo non-surgical treatment, while not directly parallel to active surveillance, may provide unique insights for developing rational inclusion criteria and potentially expanding these criteria. This study compares the survival outcomes of non-surgical and surgical treatment in patients with thyroid papillary carcinoma based on the SEER database and explores whether surgery can achieve significant survival benefits in PTC patients with larger tumor diameters, to evaluate the feasibility of expanding the active surveillance threshold.

## Materials and methods

### Data source

The SEER program was established in 1973 and is supported by the National Cancer Institute (NCI) of the USA (12). Nowadays, the SEER Program captures reported cancer cases from 19 U.S. geographic areas, representing about 30% of the population. The SEER17 Regs Custom Data with additional treatment fields (Nov 2021 submission) was utilized spanning 2000 through 2019 and weaned with SEER*Stat v8.4.0. Because the SEER database is a large, population-based cancer registry with patient-level data, results can be better extrapolated to the general population than studies made in single centers.

### Patient selection

The extraction criteria were as follows: “Primary Site = C73.9-Thyroid gland” and ICD-03 histology comprised 8050 (PTC NOS), 8260 (PTC), 8340 (follicular variant of PTC), 8341 (PTC, microcarcinoma), and 8343 (PTC, encapsulated). To mirror active surveillance criteria, patients were required to be N0 and M0 based on clinical or pathological criteria. The exclusion criteria were as follows: (1) non-histological diagnosis; (2) Survival time unknown; (3) Tumor size unknown; (4) Marriage status unknown; (5) Unknown if surgery performed. The variables extracted from eligible cases included the following: age at diagnosis, sex, race/ethnicity, year of diagnosis, follicular variant, multifocality, tumor size, radiotherapy recode, chemotherapy recode, distant metastases record, number of lesions, follow-up months, SEER cause-specific death classification, and vital status recode (study cutoff used).

### Variables collected

All eligible PTC patients were divided into Surgical and No-surgical cohorts based on whether surgery was performed and then matched with propensity scores to obtain a more comparable cohort. The following parameters were collected from the sample: age group (<55/≥55), sex (Female/Male), marital status (yes/no), race (White/Asian/Black/Other), follicular variant (yes/no), tumor size(,cm) (0-1/1.1-2.0/2.1-4.0/>4), status (alive/dead), Cancer-Specific death (yes/no), multifocality (yes/no), radioactive iodine (yes/no), chemotherapy (yes/no). The primary outcomes investigated were disease-specific survival (DSS) and overall survival (OS). DSS was classified on the basis of available death certificate information using SEER-defined variables. OS was defined as the time from diagnosis until death or last follow-up.

### Propensity score-matching

The PSM analysis was performed to balance baseline confounding factors between patients with Surgical and those with No-surgical (13). The “Matchit” package in R studio was used to match the propensity score between cohorts, and the matching approach was set as the nearest neighbor algorithm with a matching ratio of 1:1 and a caliper value of 0.03 (14). Validation of PSM was achieved by comparing the Surgical and No-surgical groups for each observed variable before and after PSM. Continuous variables were compared with unpaired student t-tests, and categorical variables were compared with χ^2^-tests.

### Survival analysis

Overall survival (OS) and disease-specific survival (DSS) was estimated using the Kaplan–Meier method. The difference in median survival between surgical groups was examined using the log-rank test. Cox proportional hazards models (with ties handled by Breslow approximation) were fitted for all predictor variables using the forward-stepwise-selection procedure from Ekman et al (15). This procedure generated 11 models, from a null model with no factors to a full model with all 10 factors. Thus, we used an information-theoretic framework to find the best explanatory models from the full set (16, 17). Specifically, the corrected Akaike Information Criterion (AICc) was calculated for each model, which indexes the amount of information provided by a model whilst penalizing it for being overloaded with factors. Data analysis was performed using R (version 4.2.2) with library MuMIn.

### Statistical analysis

In this study, continuous variables with a normal distribution were expressed as mean and standard deviation (SD), and nonnormally distributed variables as median and interquartile range (IQR). The student’s t-test (normally distributed) or Mann-Whitney U-test (nonnormally distributed) was used to compare continuous variables. Categorical variables were presented as frequencies and percentages (%) and analyzed using Fisher’s exact test or Pearson *x^2^* test. P-value <0.05 was considered statistically significant. Analyses and visualizations were carried out using R studio version 4.2.2 (http://www.r-project.org).

## Results

### Selection of study cohort and propensity score-matching

**Figure 1:**
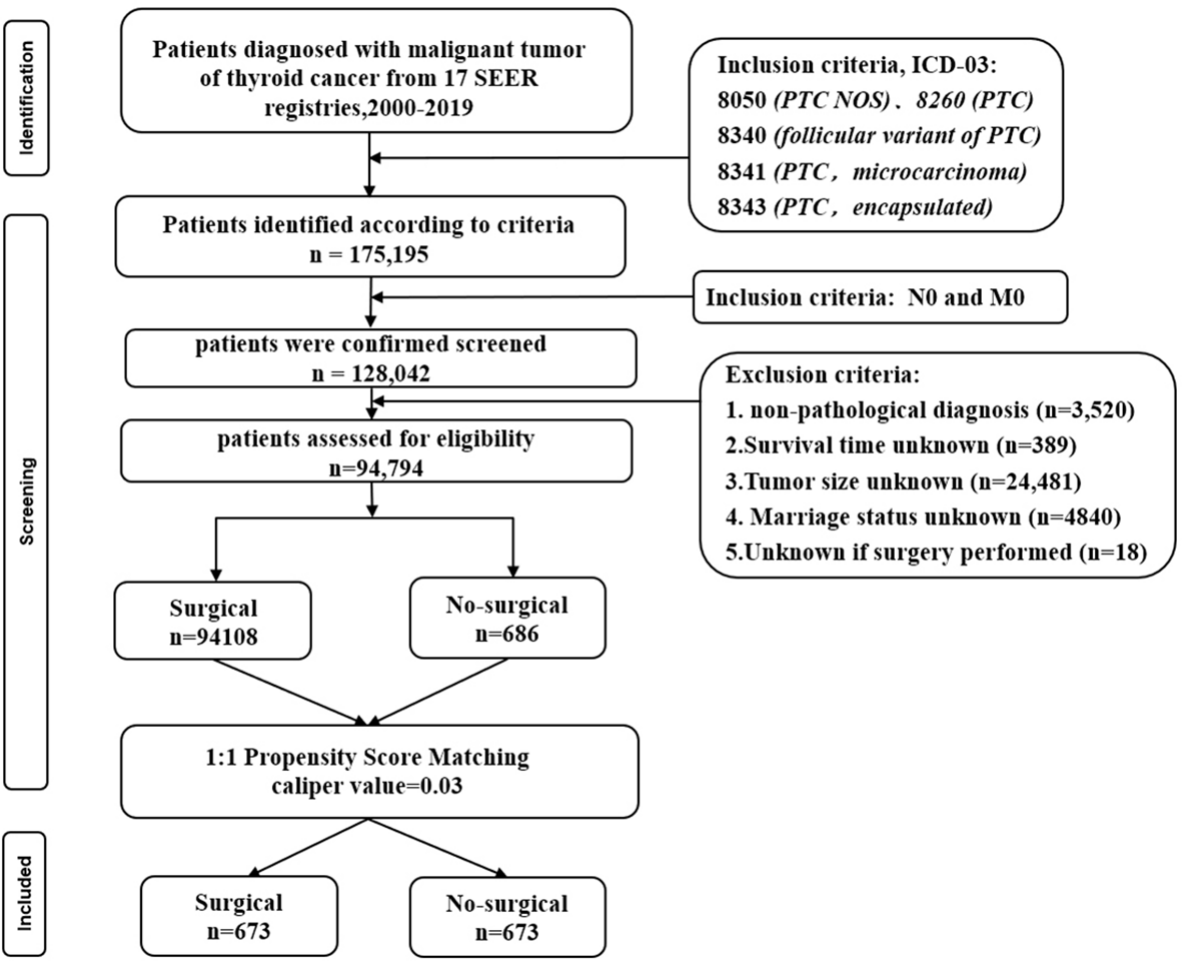
Flow diagram presenting the screening process in the SEER database.

**Figure 2:**
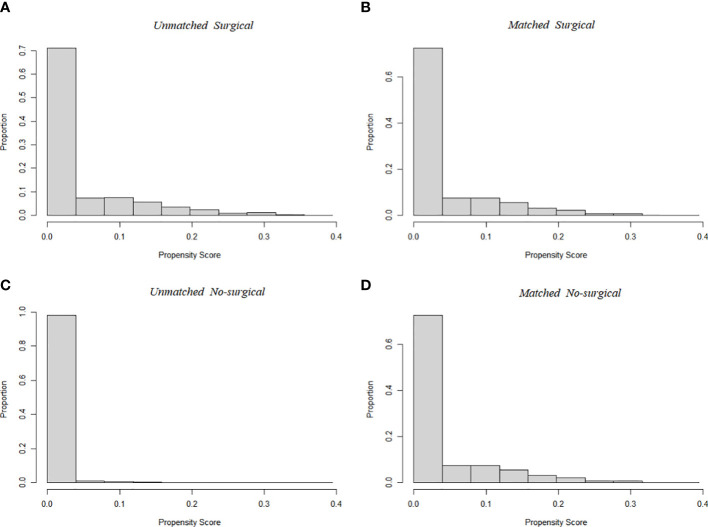
Propensity score distribution between Surgical (**A**, before PSM; **B**, after PSM) and No-surgical group (**C**, before PSM; **D**, after PSM). Compared to the unmatched sample, the matched sample exhibited nearly complete overlap, indicating better comparability between the two cohorts.

A total of 94,794 patients were extracted from the SEER database for inclusion in this study. The detailed flow diagram showing the patient inclusion and exclusion criteria process in the SEER database is shown in **Figure 1**. Of these patients, 94108 (99.3%) underwent Surgical of the PTC, while the remaining 686 (0.7%) did not. The largest proportion of PTC was 0-1cm (45.1%), and PTC smaller than 2cm accounted for 73.8% of the total population. The propensity-score distribution after matching is basically the same between the two groups, as shown in **Figure 2**.

**Table 1** shows the demographic and clinical characteristics of patients in the full and reduced datasets. The comparisons without PSM show that baseline characteristics were significantly unbalanced between the two groups for multiple covariates (P<0.05). PSM adjustment resulted in 1364 patients enrolled, 673 in each group. There were no significant differences between groups in covariates after PSM. (P>0.05) Thus, PSM appears to minimize potential confounds.

**Table 1:** Statistical results and clinicopathological characteristics of patients in the SEER database before and after propensity score matching.

Characteristic	Pre–PSM				Post–PSM			
	Overall	Surgical	No-surgical	P-value	Overall	Surgical	No-surgical	P-value
	(N=94794)	(n=94108)	(n=686)		(n=1346)	(n=673)	(n=673)	
**Age group**				<0.001				0.783
<55	55761 (58.8%)	55468 (58.9%)	293 (42.7%)		576 (42.8%)	285 (42.3%)	291 (43.2%)	
≥55	39033 (41.2%)	38640 (41.1%)	393 (57.3%)		770 (57.2%)	388 (57.7%)	382 (56.8%)	
**Sex**				<0.001				0.906
Female	74722 (78.8%)	74252 (78.9%)	470 (68.5%)		927 (68.9%)	462 (68.6%)	465 (69.1%)	
Male	20072 (21.2%)	19856 (21.1%)	216 (31.5%)		419 (31.1%)	211 (31.4%)	208 (30.9%)	
**Marital status**				<0.001				1
Married	62542 (66.0%)	62158 (66.0%)	384 (56.0%)		753 (55.9%)	376 (55.9%)	377 (56.0%)	
Not married	32252 (34.0%)	31950 (34.0%)	302 (44.0%)		593 (44.1%)	297 (44.1%)	296 (44.0%)	
**Race**				<0.001				0.982
White	77445 (81.7%)	76926 (81.7%)	519 (75.7%)		1036 (77.0%)	521 (77.4%)	515 (76.5%)	
Asian	9954 (10.5%)	9840 (10.5%)	114 (16.6%)		210 (15.6%)	103 (15.3%)	107 (15.9%)	
Black	5939 (6.3%)	5903 (6.3%)	36 (5.2%)		68 (5.1%)	33 (4.9%)	35 (5.2%)	
Other	1456 (1.5%)	1439 (1.5%)	17 (2.5%)		32 (2.4%)	16 (2.4%)	16 (2.4%)	
**Follicular variant**				<0.001				1
yes	31678 (33.4%)	31612 (33.6%)	66 (9.6%)		132 (9.8%)	66 (9.8%)	66 (9.8%)	
no	63116 (66.6%)	62496 (66.4%)	620 (90.4%)		1214 (90.2%)	607 (90.2%)	607 (90.2%)	
**Size, cm**				<0.001				0.998
0-1	42739 (45.1%)	42576 (45.2%)	163 (23.8%)		324 (24.1%)	161 (23.9%)	163 (24.2%)	
1.1-2.0	27206 (28.7%)	26984 (28.7%)	222 (32.4%)		439 (32.6%)	219 (32.5%)	220 (32.7%)	
2.1-4.0	18608 (19.6%)	18410 (19.6%)	198 (28.9%)		389 (28.9%)	196 (29.1%)	193 (28.7%)	
>4.0	6241 (6.6%)	6138 (6.5%)	103 (15.0%)		194 (14.4%)	97 (14.4%)	97 (14.4%)	
**Status**				<0.001				0.909
Alive	87106 (91.9%)	86673 (92.1%)	433 (63.1%)		867 (64.4%)	435 (64.6%)	432 (64.2%)	
Dead	7688 (8.1%)	7435 (7.9%)	253 (36.9%)		479 (35.6%)	238 (35.4%)	241 (35.8%)	
**Cancer-Specific death**				<0.001				0.854
yes	1221 (1.3%)	1147 (1.2%)	74 (10.8%)		131 (9.7%)	64 (9.5%)	67 (10.0%)	
no	93573 (98.7%)	92961 (98.8%)	612 (89.2%)		1215 (90.3%)	609 (90.5%)	606 (90.0%)	
**Multifocality**				<0.001				0.849
yes	17794 (18.8%)	17626 (18.7%)	168 (24.5%)		328 (24.4%)	162 (24.1%)	166 (24.7%)	
no	77000 (81.2%)	76482 (81.3%)	518 (75.5%)		1018 (75.6%)	511 (75.9%)	507 (75.3%)	
**Radioactive iodine**				<0.001				1
Absent	38194 (40.3%)	38176 (40.6%)	18 (2.6%)		36 (2.7%)	18 (2.7%)	18 (2.7%)	
Present	56600 (59.7%)	55932 (59.4%)	668 (97.4%)		1310 (97.3%)	655 (97.3%)	655 (97.3%)	
**Chemotherapy**				<0.001				1
yes	233 (0.2%)	215 (0.2%)	18 (2.6%)		25 (1.9%)	13 (1.9%)	12 (1.8%)	
no	94561 (99.8%)	93893 (99.8%)	668 (97.4%)		1321 (98.1%)	660 (98.1%)	661 (98.2%)	

### Survival analysis outcomes in patients after propensity score-matching

In this study, an IT-AIC approach was used to estimate the effects of Surgical in a multivariate setting, and to identify additional prognostic factors that could enhance Surgical selection (18). According to AICc, there was no single definitive model that could best explain overall survival (**Table 2**). The highest-ranked model in OS included nine factors and 31% was probably the best approximation model considered (**Table 2A**). There were six factors in the top-ranked model in DSS, and 18% may be the best approximation model available (**Table 2B**). In OS these models suggest that the following factors are informative for predicting survival: age, chemotherapy, follicular variant, marital status, multifocality, race, sex, tumor size and surgery. However, radioiodine was not a significant predictor of OS after evaluation of the best model selected by a forward stepwise selection method based on the Adjusted Akaike Information Criteria (AIC) ranking (**Table 2A**). It appears; however, the following factors were found to be effective predictors of survival in the DSS model: age, chemotherapy, multifocality, radioactive iodine, tumor size, and surgery. After assessing the optimal model selected by the forward-stepwise selection method using the corrected AIC rankings, the follicular variant, marital status, race, and sex did not significantly contribute to the predictive accuracy of DSS (**Table 2B**).

**Table 2:** This collection of models has been generated using the forward-stepwise selection approach and sorted based on the corrected Akaike Information Criterion (AIC) rankings.

A														
age	chm	fll	mrt	mlt	rac	sex	siz	sur	rai	Df	LL	AICc	ΔAIC	AICcW
										13	-3038.97	6104.71	0.00	0.31
										14	-3038.00	6104.91	0.20	0.28
										12	-3052.93	6130.52	25.81	0.00
										9	-3061.96	6142.31	37.60	0.00
										8	-3068.30	6152.90	48.19	0.00
										5	-3074.04	6158.21	53.50	0.00
										4	-3100.01	6208.11	103.40	0.00
										3	-3103.39	6212.82	108.11	0.00
										2	-3106.17	6216.37	111.66	0.00
										1	-3113.77	6229.55	124.84	0.00
										0	-3230.64	6461.28	356.56	0.00
B														
age	chm	mlt	rai	siz	sur	fll	mrt	rac	sex	Df	LL	AICc	ΔAIC	AICcW
										8	-735.70	1488.58	0.00	0.18
										9	-735.12	1489.72	1.14	0.10
										10	-734.11	1490.06	1.48	0.09
										7	-739.50	1493.90	5.33	0.01
										13	-732.84	1494.79	6.21	0.01
										14	-731.90	1495.41	6.83	0.01
										4	-769.09	1546.50	57.92	0.00
										3	-772.08	1550.35	61.77	0.00
										2	-832.92	1669.93	181.35	0.00
										1	-854.11	1710.24	221.66	0.00
										0	-901.25	1802.51	313.93	0.00

The shaded boxes in the model represent the included factors, with darker shadows indicating the most accurate approximations. Df denotes the number of parameters, LL stands for log-likelihood, and ΔAIC represents the difference in corrected AIC compared to the top-ranked model. AICcWt reflects the proportional AICc weight of the model in the entire set, providing an estimate of the likelihood that a particular model is the best one among the options. age: Age group (<55/≥55); chm: Chemotherapy (yes/no); mlt: Multifocality (yes/no); rai: Radioactive iodine (yes/no); siz: Tumor size (, cm) (0-1/1.1-2.0/2.1-4.0/>4); sur: Surgery was performed on the primary tumor (yes/no); fil: Follicular variant(yes/no); mrt: marital status(yes/no); rac: Race (White/Asian/Black/Other); sex: Sex (Female/Male). (A) OS; (B) DSS.

**Figure 3:**
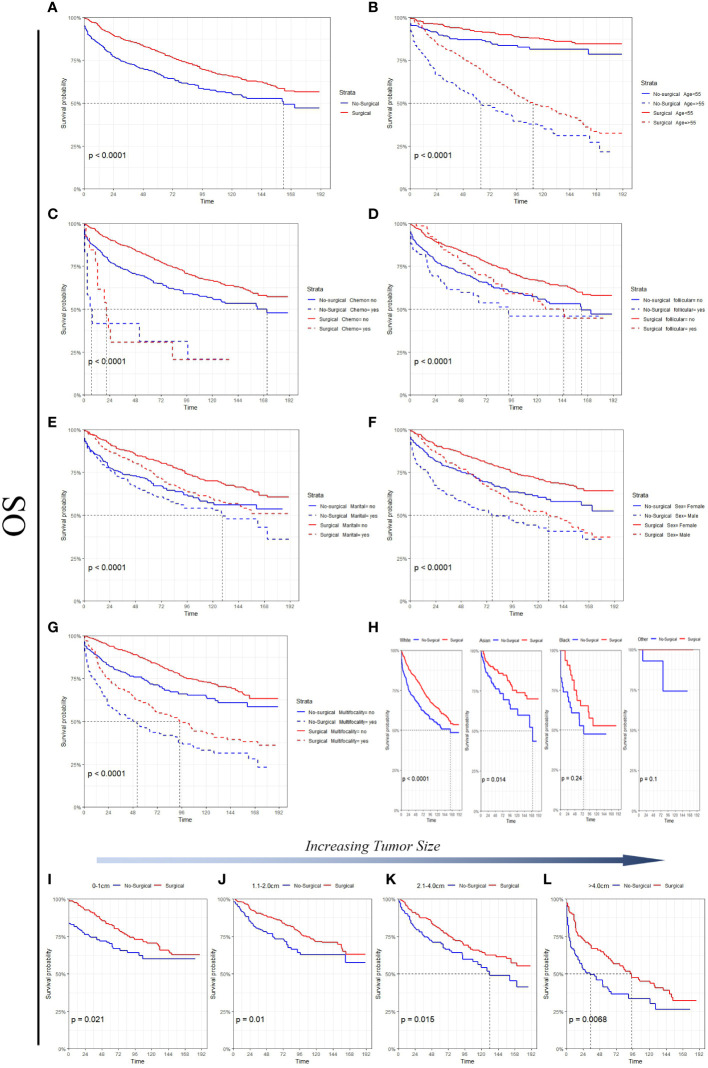
A comparison of OS for Surgical and No-surgical patients with differing tumor sizes or characteristics. **(A)** All factors; **(B)** age; **(C)** chemotherapy; **(D)** follicular; **(E)** marital status; **(F)** sex; **(G)** multifocality; **(H)** race; **(I)** tumor size: 0-1cm; **(J)** tumor size: 1.1-2.0cm; **(K)** tumor size: 2.1-4.0cm; **(L)** tumor size: >4.0cm.

**Figure 4:**
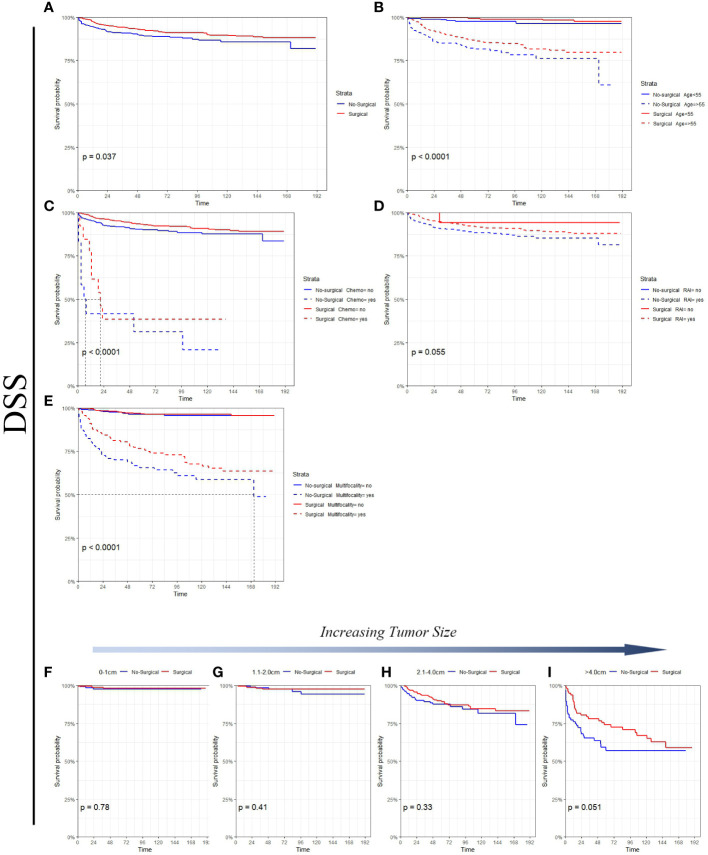
DSS comparison of surgical and non-surgical patients with different tumor sizes or characteristics. **(A)** All factors; **(B)** age; **(C)** chemotherapy; **(D)** radioactive iodine; **(E)** multifocality; **(F)** tumor size: 0-1cm; **(G)** tumor size: 1.1-2.0cm; **(H)** tumor size: 2.1-4.0cm; **(I)** tumor size: >4.0cm.

**Figure 3** Survival analysis suggested that in the non-surgery group, the 5 - and 10-year OS rates were approximately 82.9% and 70.5%, respectively, for the tumor size (0-1 cm) and 73.3% and 62.8%, respectively, for the tumor size (1.1-2.0 cm). **Figure 4** Survival analysis shows that the 5-year and 10-year DSS for tumor size (0- 1 cm) in the non-surgical group are both 97.6%, and the 5-year and 10-year DSS for tumor size (1.1- 2.0 cm) are about 97.5% and 94.1%, respectively. Detailed data are presented in **Table 3**. As well as age, gender, race, follicular variation, and multifocality, the surgical group showed a better prognosis (all P<0.01).

**Table 3:** Prognosis estimation based on survival analysis for different types of patients.

Categories	OS		DSS	
	5-year	10-year	5-year	10-year
Size, cm: 0-1				
Surgical	82.9%	70.5%	98.0%	98.0%
No-surgical	70.1%	60.1%	97.6%	97.6%
Size, cm: 1.1-2.0				
Surgical	86.4%	71.7%	97.6%	97.6%
No-surgical	73.3%	62.8%	97.5%	94.1%

OS, overall survival; DSS, disease-specific survival.

**Figure 5:**
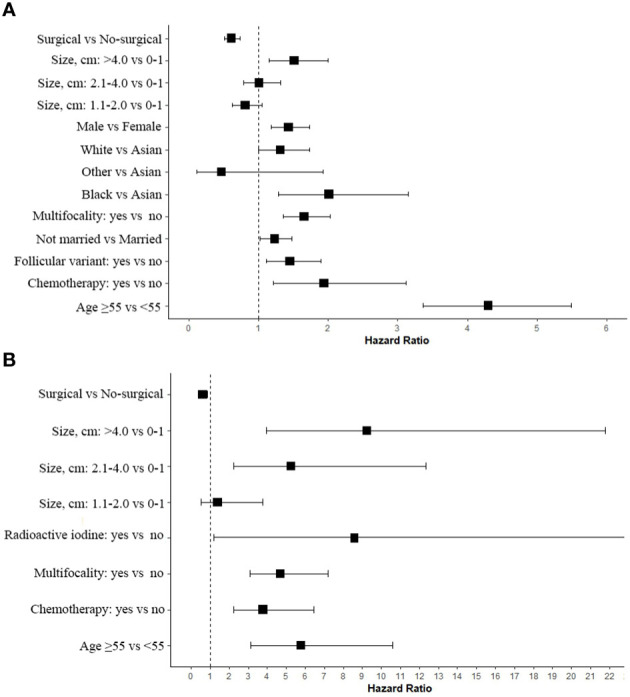
Estimated Cox proportional hazard ratios with 95% confidence intervals based on the full model average. In the plot, a dashed line indicates that the hazard ratio is equivalent to 1 (HR = 1). **(A)** OS; **(B)** DSS.

Based on AICc weighting, we estimated hazard ratios for each of these factors by averaging the estimates from each model in the confidence set (**Figure 5**). It is implied by these estimates that patients who receive surgical treatment have improved survival rates (**Figure 5**). It was certain that Surgical would play a significant role in determining the optimal model (**Table 2**). In OS, age group greater than 55 years was the most important factor affecting tumor negatively survival (**Figure 5A**). Among the factors negatively affecting DSS, A tumor with a diameter greater than 4cm was the most significant (**Figure 5B**). There was a negative impact of tumor multifocality and chemotherapy on the survival of papillary thyroid carcinoma, both in terms of OS and DSS. Overall, enlargement led to a progressively greater difference in thyroid cancer-related mortality between nonsurgical and surgical patients.

## Discussion

In recent years, with the increasing incidence of well differentiated thyroid cancer (WDTC) but stable mortality rates, overtreatment of papillary thyroid carcinoma (PTC) has become a major concern (19). Therefore, more and more studies are exploring the feasibility of AS in the treatment of PTC. In this study, using propensity score matching (PSM), we elucidated the clinical and pathological differences between the surgical and non-surgical groups and evaluated the survival prognosis based on data from 94,794 PTC patients in the SEER database. In this study, we focused on the collective impact of tumor size and age on the survival rates of a large group of non-surgically treated patients with papillary thyroid carcinoma. Given the relevance of this factor in active surveillance decision-making, we placed particular emphasis on this factor. The increase in tumor diameter is independently associated with cancer-specific death risk, and papillary thyroid carcinomas larger than 2.0cm demonstrate a relatively greater degree of hazard in survival analysis. Compared to surgical patients, we have demonstrated that non-surgical patients exhibit roughly similar disease-specific survival rates when they are young, but the risk of thyroid cancer-related death increases in old age. For decades, surgery has been the central strategy for the treatment of PTC, especially for tumors with invasive histology and regional lymph node metastasis. However, for the cohort studied here, relative benefits are not meaningful before considering patients who are older or have larger tumors than those in previous studies. The inertia of PTC suggests that survival differences may be difficult to distinguish in younger or smaller diameter tumors and longer follow-up is needed to observe any differences. Just as with active surveillance, we limited our study to cases without regional and distant metastasis, and our conclusion is that late-stage patients are expected to fare worse if they do not receive surgical treatment. It is worth noting that although non-surgical patients may not have received modern active surveillance protocols, they still have good outcomes, and similar or better outcomes are possible if there is strict monitoring and clear parameters to control the indications for surgery.

Active surveillance has been used as a treatment modality in papillary microcarcinoma of the thyroid and has been adapted from similar management strategies for indolent cancers such as prostate cancer, and has been proven to be effective. Studies of over 2,000 patients in Japan, Korea, and the United States have undergone active surveillance (7, 20–22), so far, no patient who has undergone active surveillance has died from PTMC, indicating that delaying surgery does not affect the prognosis of PTMC. Although low-risk PTMC has been included in management guidelines in Japan and the United States in 2010 and 2015, respectively, long-term follow-up data on patients enrolled in active surveillance are still limited. Therefore, some clinicians or patients may be unwilling to accept it. Our study results also indicate that there was no significant difference in DSS between surgical and non-surgical groups in patients with thyroid papillary carcinoma of 0-1.0cm. This result is consistent with the consensus statement of the Thyroid Cancer Management Guidelines for small papillary microcarcinomas by the Japanese Society of Endocrine Surgery (23). This suggests that AS is a feasible management strategy.

Our data suggest that there is no significant difference in the risk of tumor-specific mortality between 0-1.0cm and 1.1-2cm papillary thyroid carcinoma within 5 years, and the risk of survival accumulates gradually with tumor growth until the risk significantly increases at around 10 years. We anticipate that the true active surveillance cohort will reflect or refine our observation findings from the nonoperative cohort. Over the long term, increasing age is associated with higher risk for PTC and remains a prognostic covariate unique to the staging system of thyroid cancer (24). The reasons may include decreased uptake or response to RAI, elevated levels of thyroid-stimulating hormone, and decreased immune system function, among others (25–27). According to the Japanese model of monitoring patients, disease progression seems to decrease with age (11). Age was the most significant negative factor for OS mortality in our results. This may be due to patients actively choosing to participate in close monitoring of a cancer with very low risk and undergoing curative surgery for progression. Compared to this, non-surgical patients in SEER may be too frail or unable to undergo a series of larger surgeries, yet these patients are considered higher risk. In addition, our study results show that RAI is also an important negative factor affecting the DSS of PTC, as the patients we included were N0M0. This is consistent with a study published in New England Journal of Medicine: no RAI treatment is not inferior to low-dose RAI treatment, with fewer adverse reactions (28).

## Limitations

Firstly, as a retrospective study, there are inherent biases and uncontrollable confounding factors. These include its retrospective nature, potential coding errors in large registries, inability to confirm histology or N0 status in non-surgical patients, and lack of growth kinetic details involving local, regional, or distant spread. Small survival differences between surgical and non-surgical methods may exist in younger or smaller tumors, but this would require larger cohorts, more events, or longer follow-up to appreciate. Another explanation for the increased mortality among non-surgical patients involves attribution bias, whereby patients diagnosed with thyroid cancer may be incorrectly attributed to dying from thyroid cancer, as seen in some studies regarding active surveillance for prostate cancer (29). Additionally, elderly patients are more likely to have comorbidities that lead to non-cancer-related deaths, making it particularly prone to coding errors. Another potential confounding factor affecting survival rates is the registration record interval: because SEER only tracks whether surgery was performed within one year after diagnosis, some non-surgical cases may have undergone surgery after this period (30). These biases may have a potential impact on our conclusions.

## Conclusion

We have highlighted the impact of tumor size and age on the survival risk of non-surgical papillary thyroid carcinoma. Our findings suggest that non-surgery may lead to a continuous increase in survival risk, and active surveillance may be appropriate for 0-1.0 cm papillary thyroid carcinoma. For larger tumors, non-surgical methods as a substitute for surgery may be a potentially reasonable option, especially for young patients. However, the limitations of this study must be acknowledged; therefore, more prospective randomized controlled trials with large samples are warranted to further confirm these findings.

## Data availability statement

Publicly available datasets were analyzed in this study. This data can be found here: SEER.

## Author contributions

Conceptualization, methodology: HZ and P-fL. Data curation, writing original draft, visualization: JB and YW. HZ was responsible for supervision. Review and revising: P-fL, JB and YW.
